# History, citoarchitecture and neurophysiology of human and non human primates’ parietal lobe: A review

**DOI:** 10.1590/S1980-57642010DN40300005

**Published:** 2010

**Authors:** Tales Alexandre Aversi-Ferreira, Mariana Ferreira Pereira de Araújo, Danielly Bandeira Lopes, Hisao Nishijo

**Affiliations:** 1 System Emotional Science, Graduate School of Medicine and Pharmaceutical Sciences, University of Toyama, Sugitani 2630, Toyama, Japan.; 2 Laboratory of Neurosciences and Behavioral of Primates (NECOP), Department of Nursing, Institute of Biological Sciences (ICB), Federal University of Goiás (UFG), Goiânia GO, Brazil.

**Keywords:** neuropsychology, parietal lobe, gnosia, praxia

## Abstract

This strict localizationism had and still has its importance for the development of Neurosciences, since the analysis of changes in mental processes resulting from brain damage became the basis for understanding the brain organization. The human parietal cortex is a highly differentiated structure, consisting of citoarchitectonically defined subareas that are connected to other cortical and subcortical areas. Patients with lesions in the parietal cortex develop various types of neuropsychological manifestations, depending on the specific location of the lesion and the corresponding hemisphere and these lesions in this lobe do not cause modal specific disturbances. The establishment of homologies between the parietal region in humans and primates can be of great contribution in trying to unravel the various functions and complexity of this area.

## Abstract (pt)

O localizacionismo estrito teve e ainda tem a sua importância para o desenvolvimento da Neurociência, uma vez que a análise de mudanças nos processos mentais resultantes de danos cerebrais se tornou a base para a compreensão da organização cerebral. O córtex parietal humano é uma estrutura altamente diferenciada, composta por subáreas histologicamente definidas que estão ligadas a outras áreas corticais e subcorticais. Pacientes com lesões no córtex parietal desenvolvem vários tipos de manifestações neuropsicológicas, dependendo do local específico da lesão e do hemisfério correspondente, mas estas lesões não causam perturbações modais específicas. O estabelecimento de homologias entre a região parietal em seres humanos e os primatas podem ser de grande contribuição na tentativa de desvendar as diversas funções e complexidade desta área.

## A brief history of the origins of strict localizationism of brain functions

Over time, philosophers and researchers tried to associate the complex mental processes with specific locations in the brain. One of the pioneers in this field was Franz Joseph Gall, an anatomist of the early nineteenth century. He proposed, without any embasament in scientific facts that each human mental faculty is strictly located in a particular brain area that project to the skullcap, thus creating the phrenology.^1^

Perhaps the greatest exponent of brain function’s localizationism was Pierre Paul Broca (1824-1880), a French surgeon and anthropologist, born in Sant-Foy-la-Grande. As a neuroanatomist, he made important contributions to the understanding of the limbic system (rhinencephalon), became famous by the discovery of the speech motor center in the third convolution of the frontal lobe (known as Broca’s area) and by his studies with brains of aphasic patients, particularly the brain of his first patient in the Bicêtre Hospital in Paris, who was nicknamed “Tan” because he could only emit the sound of that word.

Broca demonstrated in 1861 that this patient had a neurosyphillitic lesion in the posterior third of his left inferior frontal gyrus, in a post mortem analysis. In addition to other observations, he postulated that the region described was responsible for the “speech motor images”, and that lesions in this area caused a condition called aphasia.^2^

Some years later, a German neurologist, Carl Wernicke (1848-1905), discovered an area in the temporal lobe, which, when injured, led to a sensory impairment of language. The patients with such lesions were unable to recognize spoken words, even when they han an intact audition. Wernicke postulated that this area (which was named in his honor), was connected to Broca’s area, thus forming a complex system responsible for the comprehension and expression of spoken language.

These findings led neuroscientists at that time to emphatically search for specific brain regions associated with each cognitive, motor or sensory function.

The Brodmann areas,^3^ for example, are used daily in the attempt to localize brain functions. The importance of these studies was recently the subject of a publication in honor of one hundred years of Brodmann’s publication.^4^

This strict localizationism had and still has its importance for the development of Neurosciences, since the analysis of changes in mental processes resulting from brain damage became the basis for understanding the brain organization. It is also fundamental to understanding the evolutionary aspects of the neural system.^5^ However, although some authors still disclose this system of strict location of brain functions, so important in a number of electrophysiological studies, other authors think that the neural system works as an orchestra, where several areas effectively engage to generate a response more or less complex.^1^

The need to understand and determine the development of human mental activity processes concerned several researchers over time. The scientific psychology focused on the description of the human activity and the exploration of structures involved in complex mental operations, such as perception, memory, speech, movement and action, as well as the development of these structures in ontogenesis.

In modern clinical neurology and neurosurgery it was possible to study and relate behavioral changes with localized brain lesions. To contribute with such studies, a new branch of science, called neuropsychology, emerges in the early twentieth century, defined by Luria^1^ how as “the science of brain organization of human mental processes” (p.26), whose specific objective is to “research the role of individual brain systems in complex forms of mental activity “(p.04). Damasio^6^ mentions that the purpose of the neuropsychological approach is to explain what are the relationships of certain cognitive operations with the neural systems and their components, noting that neuropsychology should not deal with the discovery of the brain localization of a given symptom or syndrome.

Alexander Romanovich Luria (1902-1977), a prestigious Russian neuropsychologist, was a critic of Pavlov, which earned him the expulsion of the Russian scientific society until Stalin’s death, developed numerous studies involving the relation of local brain lesions and behavioral changes.

During the Second World War, Luria worked in Kisegach with patients with brain tumor or lesions. He related these diseases with their effects on cognition, which allowed him to stablish the scientific foundations of neuropsychology. Through tests on the patients’ psychological expression, Luria could predict the cognitive disorders and diagnose the precise location of tumors for surgical intervention.

Currently, neuropsychological investigations include clinical and behavioral analysis and neuropsychological tests. However, other techniques were developed and are of great help in studies involving mental activity and brain structures. Among these techniques are Computerized Tomography (CT), Functional Magnetic Resonance Imaging (fMRI), Single Photon Emission Tomography (SPECT) and Positron Emission Tomography (PET). Of these, the neuroimaging techniques most used in neuropsychological studies are fMRI and PET.

## The functional units described by Luria and mental processes

According to Luria,^1^ the human mental processes, since they are not strictly located in specific brain areas, take place with the participation of several brain structures acting in concert, each contributing to the organization of these processes.

In general, according to Luria’s hypotheses, a human brain is composed of three basic functional units, all of them involved in any mental activity. The first functional unit regulates sleep and wakefulness, the second is responsible to obtain, process and store information about the environment and the third is involved in programming, regulating and verifying the mental activity. Accordingly, to carry out its general survival functions, the beings endowed with neural systems need to be “awake” and alert to receive and capture information from the environment, and then use this information to produce and evaluate responses.

Each functional unit has a hierarchical structure, consisted of three cortical areas. The primary afferent (projection) area has large granular afferent neurons that receive the pulse; the secondary area (projection-association) processes the information that arrive through small pyramidal neurons, which receive the modified pulse from the primary afferent areas and associate initially similar information through large lateral connection; the tertiary area (or association area) is formed by small granular and pyramidal neurons without specific modal organization and there is an overlap and participation of several brain modalities. Association areas are the last to develop through evolution, so they are more complex in humans, with comparatively higher mass and cell number.

The tertiary areas allow abstract reasoning and activities, being responsible for the humans’ complex functions.^1^

The posterior tertiary zones are formed predominantly by cells of layers II and III of the cortex, which have the role of integrating the stimuli that come from different analyzers, generating the multimodal character of this region. The main tertiary areas in the human cortex are Brodmann areas 5, 7, 39 and 40 (the upper and lower areas of the parietal region), areas 21 (temporal region), 37 and 39 (temporoparietal occipital region) and areas 9, 10 and 46 (pre-frontal region).^3^

## Citoarchitecture of the human parietal cortex

A faithful interpretation of the higher mental processes also depend on the knowledge of cellular and fibrous structures of the cerebral cortex, such as the cortical cytoarchitecture.

The human parietal cortex is a highly differentiated structure, consisting of citoarchitectonically defined subareas that are connected to other cortical and subcortical areas.^7-9^

The inferior parietal cortex (IPC) integrates several modalities (somatosensory, visual and auditory) and plays an important role in higher cognitive functions.^8^

**Figure 1:**
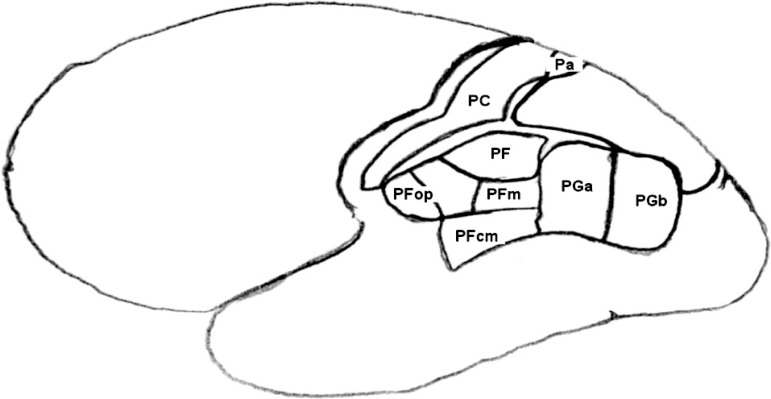
Scheme of human brain indicating the parietal lobe areas (based in Casper et al.)^8^.

In a *postmortem* study with ten human brains, the citoarachitectonic edges of the IPC were outlined using an observer-independent technique, which produced a 3-D map that correlated with data from functional imaging studies. In this study, seven areas were found in the inferior parietal cortex, being five in the supramarginal gyrus (PF, PFmc, PFm, PFop and PFt) (Figure 1) and two in the angular gyrus (PGa, PGp) (Figure 1). Those gyri are located, respectively, at Brodmann areas 40 and 3. Such citoarchitectonic design cannot be delineated by macroanatomical boundaries.^8^

Caspers et al.^8^ found that in the area PFop, located rostro-ventrally in the IPC, the layer II has relatively low cell density and the pyramidal cells in layer III are larger than those of layer V. The PFt, dorsal to PFop, has a higher cell density in layer II and its pyramidal cells in layer III are quite prominent.

PF is the largest IPC area, and is located caudal to PFT. Its layers have a high cell density and its granular cells of layer II interpenetrate in the small pyramidal cells of the upper part of layer III (which represents the major part of the cortical width). The layer IV presents vertical interruptions coming from pyramidal cells of layer III that extend into the upper part of the layer V.

The PFm area, located caudally to PF may be regarded as a transition zone between areas PF and PG. It shares common characteristics with PF, differing mainly in layer IV, which is clearly separated from the layers III and V, just as in PG. The PFmc lies ventral to PF and caudal to PFop. It has lower cell density than PF and PFm, and its layer II is clearly separated from layer III, which has large pyramidal cells that characterize, remarkably, the area PFmc.

The PGa is located rostrally in the caudal portion of the IPC. Its layer IV is displaced to a more superficial position, dividing the cortex into two bands with equal widths. PGp has a somewhat broader layer II than PGa. Its granular cells interpenetrate into the small pyramidal cells of the upper part of the layer III, which makes it difficult to identify the boundary between these layers. These are the main features that separate the rostral and caudal regions of the IPC.

The knowledge of cortical cytoarchitecture may be of great help when it comes to understanding the functionality of the parietal cortex and the cell types involved.

## Neuropsychology of the primate parietal lobe

Neurophysiological studies in nonhuman primates suggest similarities with some areas of the human parietal cortex,^10-14^ which makes the study of the parietal cortex homologies between human and non human primates a promising field that can help uncovering the multiple functions of this region. Several authors suggest homology between the anterior intraparietal, medial intraparietal, Brodman’s area V6a (layer V) and 34 and lateral intraparietal areas of monkeys and humans. Those areas were activated during activities that required manipulation movements and visuospatial activities (look, attention, point and grasp).^14^ In the intraparietal lateral region, some neurons were also activated by auditory stimuli, in a task in which the response was an oculomotor movement,^15^ suggesting a link between hearing and behavioral modulation of eye movement.

The area PE (Figure 1), located in the superior parietal lobule of monkeys, is regarded as a somatosensory area, and most cells of its caudal portion are activated by passive somatosensory stimulation and reaching movements.^13^ This region, however, has no somatotopic organization.

Other authors consider that this area may be involved in the representation of the body in the space.^14^ Capp et al.^16^ injected BDA (biotinylated dextran amine) in the posterior parietal cortex of *Macaca mulatta and M. fascicularis* individuals and observed the thalamo-cortical (TC) and cortico-thalamic (CT) projections to areas PE and PEa, which may represent a possible anatomical substrate for the transthalamic, multisensory and sensorimotor integration processes involving the area 5 in primates, relevant to visual guidance and reaching movements.

The area 7a, located in the posterior superior parietal lobe, is a subject of study by many authors, who observed the modulation of the reported region in Rhesus monkeys (*M. mullata*) in conditions involving spatial attention,^17^ speed variance and selectivity,^11^ combination of information from eye and hand movement^18^ and neural representation of space.^10,19^ Other studies, involving the inferior parietal lobe of rhesus monkeys, detected the activation of the same, plus the prefrontal cortex, in tasks that required working memory.^20^

Most studies involving the parietal region of nonhuman primates address the relationships between this region and visuomotor processes.^17,21-23^ Visual stimuli usually weakly activate the neurons in the intraparietal lateral region. However, a large activation can be recorded in this same area if the visual stimuli have abrupt onsets or if they are embebbed in a behavioral context.^23^

Pitzalis et al.^21^ believe, based on the similarity of position, visuotopic organization and relationship with neighboring extraestriate visual areas, that there may be an homology between the human Brodman’s area V6 and the medial and dorsomedial areas of New World primates.

Recently, the use of capuchin monkeys (*Cebus libidinosus*, C.l.),^24^ has increased and became an important tool in Neuroscience. These New World primates have a large geographical distribution, being found from Colombia and Venezuela all the way to northern Argentina. They inhabit tropical, subtropical and riverside forests, as well as Cerrado and semi-arid regions in Brazil.^25^

The studies with capuchin monkeys involve behavior,^25-27^ anatomy,^28-37^ physiology,^38^ tool use,^39-41^ cortical anatomy,^5^ encephalic index^42^ and memory.^43^ These monkeys have a high cognitive capacity and display an immense ability to handle tools for obtaining food and for amusement.^26,27^ Their behavior, memory, tool use abilities and encephalization index are close to those observed in chimpanzees. Moreover, *Cebus*, humans and Old World primates have the same basic neural substrate for memory, and learning tests indicate a long term convergence of the development of these species.^47^

**Figure 2:**
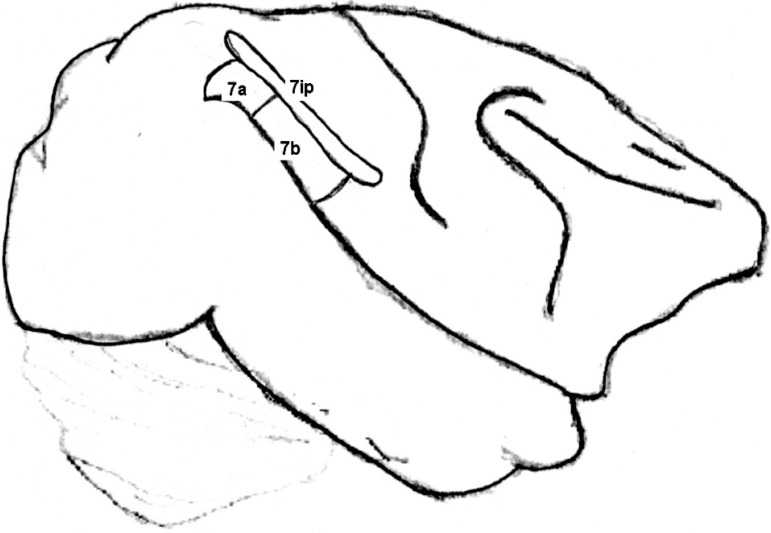
Cebus parietal cortex areas (according to Leichnetz)^37^.

Additionally, according to Leichnetz,^37^ their medial posterior parietal cortex cortical and subcortical afferents and efferents connections with the ipsilateral medial bank of the intraparietal sulcus and adjacent superior parietal lobule, inferior parietal lobule (area 7a), lateral bank of the intraparietal sulcus (area 7ip)(Figure 2), caudal parietal operculum, dorsal bank of the caudal superior temporal sulcus (visual area), and medial prestriate cortex (including visual area and caudal medial lobule), are similar to the ones found in Rhesus and chimpazees.

The aspects cited above indicate that capuchin monkeys can be used in neural tests due to their similarities with Old World primates and humans. In many cases, they can represent a better alternative, since the they are medium weight primates (and therefore less expensive to maintain) that can be found in extensive areas in south and central america.

## Neuropsychology of the human parietal lobe

The parietal lobe is considered one of the most complex regions in the human brain because, unlike the occipital and temporal lobes, which have specific modal functions (vision and hearing, respectively), it is an association area, responsible for organizing simultaneous syntheses.^1^

Due to its modal characteristics, the parietal cortex is considered a challenge to neuroimage techniques,^9^ which are the main tools used to map the human brain. Therefore, a better understanding of the neuropsychological aspects related to the parietal lobe can be achieved through the study and analysis of patients with lesions in this brain region.

Patients with lesions in the parietal cortex develop various types of neuropsychological manifestations, depending on the specific location of the lesion and the corresponding hemisphere.^1^ According to Luria,^1^ lesions in this lobe do not cause modal specific disturbances. So, vision, hearing and tactile and kinesthetic sensibilities remain unchanged.

Agnosias, apraxia and Wernicke’s aphasia are the parietal and temporal lobe lesions’ most characteristic alterations. Agnosias are disturbances of perception regarding the loss of ability to recognize visual, auditory and somesthetic stimuli, without compromising the level of awareness and sensitivity. Apraxia is the inability to perform motor acts under an order or imitation, in the absence of a deficit in understanding, sensibility or muscle strength. Wernicke’s aphasia is characterized by impairment in language and verbal repetition, with the verbal fluency preserved.

Patients with lesions in the left inferior parietal cortex present changes in information reception and analysis, difficulties to fathom relationships in the space and difficulties in differentiating left and right. Besides, they can have constructive ataxia, semantic and amnesic aphasia, acalculia and paraphasia. The right hemisphere participates directly in perceptual processes and is responsible for more direct, visual, relations with the outside world. Lesions in the right inferior parietal cortex do not cause changes in the understanding of logical-grammatical structures or in the execution of complex mathematical operations.^1^ Furthermore, patients with lesions in this region often present disturbances in the processes of spatial gnosia and praxia not linked with the speech system. These disorders are characterized mainly by: unilateral spatial agnosia, in which the patient expresses unconsciousness of the left half of his visual field; anosognosia, in which the patients do not notice their mistakes and exhibit in a special way the symptom of lack of awareness of their own flaws; and prosopagnosia (basal portion of temporal lobe – fusiform gyrus), a disorder of face recognition.

A study carried out by Carrilho et al.^48^ involved four patients with the alien hand syndrome, a rare neurological disorder in which one hand acts involuntarily, without the patient noticing it.

Symptoms include grabbing and squeezing, touching the face or tearing the clothes involuntarily, fill the mouth with food, prevent the normal hand to do simple tasks and poke and choke their selves. The results revealed great damage in the contralateral parietal cortex, reinforcing the theory that parietal lobe lesions may play a role in the genesis of unintentional and involuntary levitation of the hand. Studies with fMRI also revealed symmetric activation of the parietal lobes in visuospatial tasks (pick up, pointing, gaze and attention) using the right hand.^14^

Another function associated with the parietal lobe is the control of attention, which involves the factors, or cognitive parameters, that determine which environmental input receives attention and which do not.^49^ There are two kinds of cognitive parameters : one known as “attention capture” (stimulus-driven/data-drive) and another resulting from the explicit will of the body (goal-directed/goal-drive).^49,50^

In a study that investigated the parameters of attention control that may be affected in patients with damage in the parietal region, normal individuals were induced into a state of neglect. Spatial neglect is characterized as a failure of the individual to explore the contralateral side of the lesion and to react to stimuli that come from this side.^51,52^ It is usually caused by damage to the right hemisphere.^1,53^ This study suggests that the parameter of “attention capture” may be impaired in patients with neglect.^49^ In a similar study, Hilgetag et al.^51^ detected phenomena commonly observed in neglect patients, and a significant improvement to targets located ipsilaterally to the lesion. Previous studies reported the involvement of the damaged parietal lobe in attention deficit when the target is located contralaterally to the lesion, thus indicating a connection of the parietal lobe in the process of selective attention.^52^

Visual attention is the ability to process, selectively, only a subset of information present in the image that falls in the retina.^54^ Visual neglect in patients with brain injuries is not related with damage in the primary visual cortex, but in regions of the contralateral parietal lobe, specifically the right inferior parietal lobule.^55,56^ In an fMRI study, activations in the left and right primary visual cortices of a patient with damage in the lower right parietal lobe were observed,^56^ indicating that the initial stages of visual processing were still intact, even in the hemisphere with parietal damage.^55^ This activity, however, was not sufficient to raise visual awareness in the neglected region.^56^

Despite all the evidences suggesting that spatial neglect is related to damage in the parietal lobe, some authors suggested that it results from damage in the superior temporal gyrus^57,58^ and subcortical structures connected to this region, such as caudate nucleus, putamen and pulvinar.^57^ Others also highlight the involvement of the right frontal lobe, cingulate gyrus,^24^ and parahippocampal region.^53,58^

Mort et al.^58^ analysed patients who had suffered stroke in the middle or posterior cerebral arteries of the right hemisphere and presented neglect. The authors showed that the angular gyrus of the inferior parietal lobe was the critical area involved in cases of stroke. On the other hand, stroke in PCa (Figure 1) involved the parahippocampal area, on the surface of the medial temporal lobe, indicating then that both regions are involved in neglect. Those results are contrary to the idea proposed by Karnath et al.,^57^ in which only the superior temporal gyrus and subcortical structures connected to it are implicated in neglect. Mesulam^53^ considers neglect a “network syndrome”, where the damage on one or more components of this network cause changes, due to the fact that these components have different physiological and anatomical relationships.

Experiments with healthy subjects performing tasks requiring mental rotation, showed a significant activation of the right superior parietal lobe, more intensely in the intraparietal sulcus,^59^ and of the primary and sencondary sensorimotor areas.^60^ Wolbers et al.^61^ verified the activation, in humans, of the contralateral superior parietal lobe when mental rotation tasks were combined with motor images of hands.

Patients with lesions in the left parieto-occipital region (left angular gyrus), usually show symptoms that characterize the Gerstmann syndrome, such as spatial disorders, constructive apraxia and inability to name fingers. Beside these, difficulties to aprehend logical-grammatical relationships and disturbances in the execution of mathematical operations are common.^1^

In a study with a patient that had damaged the left perisylvian area and presented aphasia, dyslexia and acalculia, a deficit in tasks involving numbers in verbal form (dictation and reading) or verbal responses to questions of numerical knowledge was also observed. The ability to manipulate non-verbal representations of numbers as Arabic numerals and amounts, however, was still preserved.^62^ Activities involving the overlap of calculation and language activate the left posterior segment of the intraparietal sulcus, below the left angular gyrus.^14^ These studies suggest a possible common neural network between the processes of language and arithmetic.

Cohen et al.,^62^ also observed a bilateral activation of the parietal region and adjacent areas, suggesting that the right parietal lobe plays a role in arithmetic activities. In a topographic study conducted by Simon et al.^14^ the activation was detected from the left intraparietal sulcus to the upper part of the posterior segment of the post-central sulcus; in the right hemisphere, the activation occurred in the horizontal segment of the intraparietal sulcus. These studies suggest that the right parietal lobe also plays a role in the process of calculation.

The process of mental arithmetic activities involves different regions in kids/teenagers and adults. In the later group, the left parietal cortex, along with the supramarginal gyrus and adjacent anterior intraparietal sulcus, and the occipital and left lateral temporal cortices are activated. In younger subjects, the activation occurs on the dorsal and ventrolateral prefrontal cortices, anterior cingulate gyrus, hippocampus and dorsal basal ganglia, suggesting more use of working memory, attention and declarative and procedural memory. Thus, it seems that occurs a specialization of the left inferior parietal cortex, in relation to arithmetic processes, as people get old.^63^

Dehaene et al.^64^ proposed the coexistence of three circuits in the parietal lobe associated with different arithmetic activities: a bilateral intraparietal associated to a quantity system, a region on the left angular gyrus associated with verbal processing of numbers and a system on the posterior superior parietal associated with spatial and non-spatial attention.

In a study using methylphenidate, an indirect catecholamine agonist, used to treat attention deficit disorders and hyperactivity, the working memory of patients with blood flow reduction in the dorsolateral prefrontal and posterior parietal cortices was improved,^21^ implying that the posterior parietal cortex may be involved in the attention mechanisms used in working memory.^65^ Jonides et al.^65^ also observed the activation of the posterior parietal córtex, posterior region of temporal posterior gyrus, and also, other brain areas in conditions of storage and retrieval of verbal working memory.

## Conclusions

The region of the human parietal lobe is a of intense complexity, since it is an association area formed predominantly by cells of the layers II and III of the cerebral cortex, which are responsible for the integration of various stimuli.

The neuropsychological aspects involved in the parietal cortex are diverse and varied, depending on the hemisphere involved and the affected region, because this cortical area establishes neural connections with many other cortical and subcortical regions.

Among the major neuropsychological deficits observed in the damaged parietal lobe are the visual and spatial neglect, acalculia and impairments in activities involving visuospatial tasks, arithmetic, language and working memory. Such operations also activate the intact parietal lobe, as well as other regions. The overlapping tasks involving calculation and language activate the posterior portion of the left intraparietal sulcus; tasks that require calculation also activate bilaterally the parietal areas, suggesting that there is a relationship between activities of calculation and language and that processing of calculations occur on both parietal lobes.

The establishment of homologies between the parietal region in humans and primates can be of great contribution in trying to unravel the various functions and complexity of this area. Most studies have considered homology in areas IPA, IPM, V6 and IPL, activated mainly by activities involving vision and spatial position. Most of these studies were conducted with Old World primates. However, in recent years, a New World primate, the capuchin monkey (Cebus) has been used and became a more viable alternative for studies of brain functions, since they have complex behavior and are studied as a model for tool use in human evolution.
